# Factors associated with metabolically healthy status in obesity, overweight, and normal weight at baseline of ELSA-Brasil

**DOI:** 10.1097/MD.0000000000004010

**Published:** 2016-07-08

**Authors:** Maria de Fátima Haueisen Sander Diniz, Alline Maria Rezende Beleigoli, Antônio Luiz P. Ribeiro, Pedro Guatimosim Vidigal, Isabela M. Bensenor, Paulo A. Lotufo, Bruce B. Duncan, Maria Inês Schmidt, Sandhi Maria Barreto

**Affiliations:** a School of Medicine & Hospital das Clínicas, Universidade Federal de Minas Gerais, Belo Horizonte, MG; b Hospital Universitário, University of São Paulo, São Paulo, SP; c Postgraduate Program in Epidemiology, School of Medicine, and Hospital de Clínicas de Porto Alegre, Federal University of Rio Grande do Sul, Porto Alegre, RS, Brazil.

**Keywords:** metabolically healthy obesity, metabolically healthy status, obesity, overweight

## Abstract

The primary aim of this study was to evaluate metabolically healthy status (MHS) among participants in obesity, overweight, and normal weight groups and characteristics associated with this phenotype using baseline data of Brazilian Longitudinal Study of Adult Health (ELSA-Brasil). The secondary aim was to investigate agreement among 4 different MHS criteria. This cross-sectional study included 14,545 participants aged 35 to 74 years with a small majority (54.1%) being women. Of all participants, 22.7% (n = 3298) were obese, 40.8% (n = 5934) were overweight, and 37.5% (n = 5313) were of normal weight.

Socio-demographic, behavioral, and anthropometric factors related to MHS were ascertained. Logistic regression models estimated the odds of associations. We used 4 different criteria separately and in combination to define MHS: the National Health and Nutrition Examination Survey (NHANES), the National Cholesterol Education Program (NCEP-ATPIII), the International Diabetes Federation (IDF) and comorbidities, and the agreement between them were evaluated by Cohen-kappa coefficient.

MHS was present among 12.0% (n = 396) of obese, 25.5% (n = 1514) of overweight, and 48.6% (n = 2582) of normal weight participants according to the combination of the 4 criteria. The agreement between all the 4 MHS criteria was strong (kappa 0.73 *P* < 0.001). In final logistic models, MHS was associated with lower age, female sex, lower body mass index (BMI), and weight change from age 20 within all BMI categories.

This study showed that, despite differences in prevalence among the 4 criteria, MHS was associated with common characteristics at every BMI category.

## Abstract 2

Supplemental Digital Content is available in the text

## 1 Introduction

According to the 2013 Global Burden of Disease Study, almost 2.1 billion adults in the world were obese [body mass index (BMI) ≥ 30 kg/m^2^] or overweight (BMI 25–29.9 kg/m^2^).^[1]^ Following the last 3 decades of socioeconomic and nutritional transition, approximately 70% of Brazilian adults are obese or overweight.^[2]^ Being obese or overweight contributes to the increasing public health burden of noncommunicable conditions such as cardiovascular diseases, type 2 diabetes, and cancer.^[1]^ In addition, the risk of mortality attributable to obesity is substantial, although a high BMI, which is widely used as a marker of obesity in population-based studies, does not necessarily reflect an increased fat mass.^[3–6]^ Clinical and epidemiological studies have demonstrated that obesity and being overweight are neither necessarily linked to an adverse cardiometabolic profile nor to higher chronic disease prevalence and mortality.^[3,7]^ Concerning this, the term metabolically healthy obesity (MHO) has been proposed to characterize a phenotype that is not accompanied by cardiometabolic dysfunction.^[8–10]^

The definition of MHO is controversial. Criteria vary among different authors with some of them having used the metabolic syndrome components (as hyperglycemia, dyslipidemia, high blood pressure)^[8]^ as part of the definition, whereas others have included insulin resistance and/or ultrasensitive C-reactive protein (CRP), a marker of inflammation linked to cardiovascular risk.^[10]^ Moreover, controversy still exists regarding the transitory nature of metabolically healthy status (MHS) in overweight and obese individuals.^[4,9]^ In fact, normal weight individuals can also have an unhealthy metabolic profile.^[11]^

Identifying factors associated with a high-risk metabolic profile within each BMI category is valuable, as it may have implications for population and clinical preventive strategies.^[6,9]^ In individuals of normal weight, this might be of particular interest due to the misconception of an inherently low metabolic risk associated with normal weight.

Given these points, this study aims to investigate the prevalence of the MHS by different criteria among obese, overweight, and normal weight participants and to determine socioeconomic, behavioral, and anthropometric factors associated with this phenotype at baseline in the Brazilian Longitudinal Study of Adult Health (ELSA-Brasil), a large multicentric cohort conducted in 6 Brazilian capitals. We also investigated the agreement of MHS criteria among this population.

## 2 Methods

### 2.1 Study design and population

This investigation was a cross-sectional analysis, a subproject of the Longitudinal Study of Adult Health (ELSA–Brasil), which has been described previously.^[12]^ Briefly, the baseline cohort comprises 15,105 active or retired civil servants of universities or research institutions from 6 cities in Brazil enrolled between August 2008 and December 2010, aged 35 to 74 years, mostly female (54%) middle-aged (78% aged <60 years) adults. All participants were volunteers and all 6 respective Institutional Review Boards approved this study. The quality and control of the collection and storage of data were ensured by training sessions, certifications, and renewal of certifications of those performing interviews, clinical examinations, and laboratory tests during the study protocol.^[13]^

We excluded participants who were underweight (BMI <18.5 kg/m^2^, n = 137), with missing values on BMI (n = 6), insulin (n = 11), triglycerides (TG) (n = 6), high-density cholesterol (HDL-C) (n = 1), and fasting plasma glucose (FPG) (n = 3). In addition, participants who previously underwent bariatric surgery (n = 120) and those with ultrasensitive CRP ≥ 20 mg/L (n = 276), values likely to be due to acute systemic inflammatory states were excluded. After the exclusions, data from the remaining 14,545 participants were analyzed.

### 2.2 Study protocol

Baseline data collection (standardized interviews, anthropometric, blood pressure measurements, and blood tests) were performed at the participants’ workplaces and at the 6 research centers. Race/skin color (white, nonwhite), marital status (married, not married), educational level [≥12 years (university degree); 9–11 years (high school); ≤8 years (elementary school)], premature birth (yes, no), self-rated health (very good or good/fair, poor or very poor) were self-reported. Social class (high, middle, or low) was categorized according to per capita household income. According to job activities performed, occupation was classified as routine manual (as manual workers), routine nonmanual (as administrative activities), or nonroutine and nonmanual (as teaching).^[12]^

### 2.3 Anthropometric data

Anthropometric parameters were measured using standardized and calibrated instruments, according to the study protocol. Weight (kg) and height (cm) were measured with the participant barefoot, wearing light clothes, and standing straight with the head level, using Toledo scales (to the nearest 100 g) and a stadiometer (accuracy of 0.1 cm), respectively. Waist (mid-point between lowest rib and iliac crest) and hip (area of greatest gluteal protuberance) circumference were measured by inelastic tapes (cm). The average of 2 measures was used for analyses. BMI [weight (kg)/height (m)^2^] was calculated. We used World Health Organization (WHO) criteria to stratify BMI into 3 categories (normal weight ≥18.5 to 24.9 kg/m^2^, overweight 25–29.9 kg/m^2^, obesity ≥30 kg/m^2^). Relative weight change from the age of 20 years old was calculated as (current weight: 20 years old weight/20 years old weight)∗ 100.^[12]^

### 2.4 Lifestyle/habits/mental health

Participants were classified as never, former, or current smokers and as never, moderate, or high (women: ≥ 140 g/week, men: ≥210 g/week) alcohol consumers. Leisure and transportation physical activity was verified through the long form of the International Physical Activity Questionnaire (IPAQ) and participants were categorized into 3 groups: low, moderately, or highly active, according to the sum of metabolic equivalents per week (a combination of activity type, frequency, and duration). We also categorized participants in adequately active (≥150 min/week of moderate aerobic activities or ≥75 min/week of vigorous aerobic activities) or not according to WHO criteria.^[14]^ Regular consumption (≥5 times/week) of fruits and vegetables was assessed by a Food Frequency Questionnaire and mental health (common mental disorders – yes/no) by the Clinical Interview Schedule Revised (CIS-R), both validated for Brazilian Portuguese.^[12]^

### 2.5 Laboratorial tests

Blood samples were collected after an overnight fast for FPG, total cholesterol, TG, HDL-C, and CRP. FPG was determined by hexokinase method (enzymatic colorimetric); total cholesterol by cholesterol oxidase method (enzymatic colorimetric), TG by glycerol–phosphate peroxidase; HDL-C by homogeneous colorimetric without precipitation, uric acid by uricase method (enzymatic colorimetric), all of them with an ADVIA 1200 Siemens® system (Deerfield, IL). Low-density cholesterol (LDL) was calculated by Friedewald Equation, when TG ≤400 mg/dL, or determined by homogeneous enzymatic colorimetric method without precipitation (ADVIA 1200 Siemens system), when TG > 400 mg/dL. Insulin was determined by immunoenzymatic assay (ADVIA Centaur Siemens®, Deerfield, IL); CRP, by immunochemistry through nephelometry (nephelometer BNII, Dade Behring; Siemens®). Homeostasis model assessment-insulin resistance (HOMA-IR) was calculated from FPG and insulin concentrations as [FPG (mg/dL) X 0.0555 X fasting serum insulin (mUI/L)/22.5].^[15]^ Microalbuminuria were determined by immunochemistry through nephelometry (nephelometer BNII Siemens) in a 12-hour urine sample.

All laboratory analyses were performed at a single research center (University of São Paulo).^[16]^

### 2.6 Comorbidity definitions

Arterial hypertension was defined by a self-reported medical diagnosis of hypertension, use of anti-hypertensive agents, or blood pressure ≥140/90 mm Hg at the moment of evaluation. Diabetes mellitus was defined by abnormal laboratory findings of FPG ≥ 126 mg/dL (≥7.0 mmol/L), or plasma glucose levels 2 hours after a 75 g load of anhydrous glucose ≥200 mg/dL (≥11.1 mmol/L), or hemoglobin A1c ≥6.5%, or by the use of insulin or oral/subcutaneous hypoglycemic drugs, or if there was a previous medical diagnosis of the disease (self-reported). Dyslipidemia was defined by TG ≥150 mg/dL (≥1.7 mmol/L) or HDL-C <40 mg/dL (<1.04 mmol/L in men) or <50 mg/dL (<1.30 mmol/L in women), or the use of lipid-lowering agents.^[12]^

### 2.7 Definition of metabolically healthy status

Healthy or unhealthy metabolic status was classified according to 4 commonly used criteria: those used in analyses by the National Health and Nutrition Examination Survey (NHANES)^[10]^; the metabolic syndrome criteria of the National Cholesterol Education Program (NCEP)^[17]^ and the International Diabetes Federation (IDF)^[18]^; and a comorbidities criteria. The last uses the presence of arterial hypertension, diabetes mellitus, or dyslipidemia, as defined above, as a proxy of unhealthy status (Table S1). Individuals were considered metabolically unhealthy if so classified by any of the 4 criteria, and as metabolically healthy if so considered by all 4 criteria. Three variables were created on the basis of the combination of individuals’ metabolic status and BMI (yes/no): metabolically healthy obese, metabolically healthy overweight, or metabolically healthy normal weight.

### 2.8 Data analysis

Normality of the data distribution was assessed by histograms and Shapiro–Wilk tests. Descriptive and univariate analyses (Chi-square, Mann–Whitney, and Student *t* test) were used to compare socio-demographic, current lifestyle habits, self-rated, and mental health between metabolic status groups, across BMI (obesity, overweight, or normal weight) categories. To assess the agreement between the 4 sets of MHS criteria, we used the Cohen-Kappa coefficient.

For each BMI category, logistic regression models were performed to determine associations between metabolic status (healthy or unhealthy) and the following covariates: Model I—sex and age; Model II—Model I and significant (*P* < 0.20) or poor relevant clinical/laboratorial, socio-demographic, current lifestyle, habits, self-rated, and mental health covariates on univariate analysis; Model III—significant variables on Model II along with BMI and relative weight change. Similar adjustments were performed for multiple logistic regression models with metabolic health status defined by NHANES, NCEP, IDF, and comorbidities criteria separately as dependent variables. As waist circumference is part of NCEP and IDF criteria, and the correlation between waist and BMI was strong (0.85), we decided to adjust either for BMI or WC. All the *P* values given are 2-sided with the level of significance set to *P* < 0.05, except for multiple comparisons when it was set to 0.05 divided by the number of comparisons.

We used STATA package 13.0 for these analyses.

## 3 Results

### 3.1 Prevalence of MHS according to BMI

Among the 14,545 participants, 22.7% (n = 3298), 40.8% (n = 5934), and 37.5% (n = 5313) were classified as obese, overweight, and normal weight), respectively. Sixty-six percent (9656) were married, and 52.3% (7516) were white. Frequency of arterial hypertension, diabetes, and dyslipidemia was 35.9%, 19.6%, and 47.5%, respectively. Characteristics of the study population are depicted in Table 1.

**Table 1:**
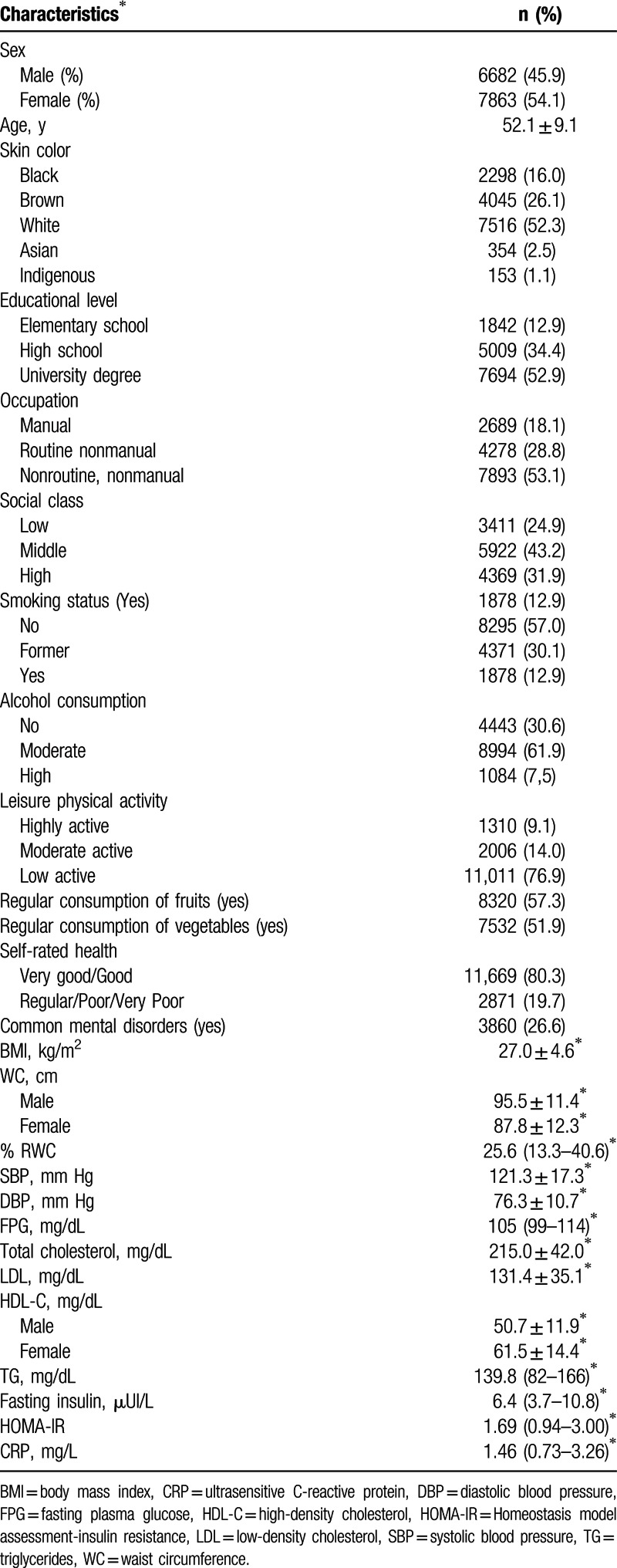
Demographic, lifestyle, self-rated and mental health characteristics, laboratorial data of study population (ELSA-Brasil 2008–2010) n = 14,545.

**Figure 1:**
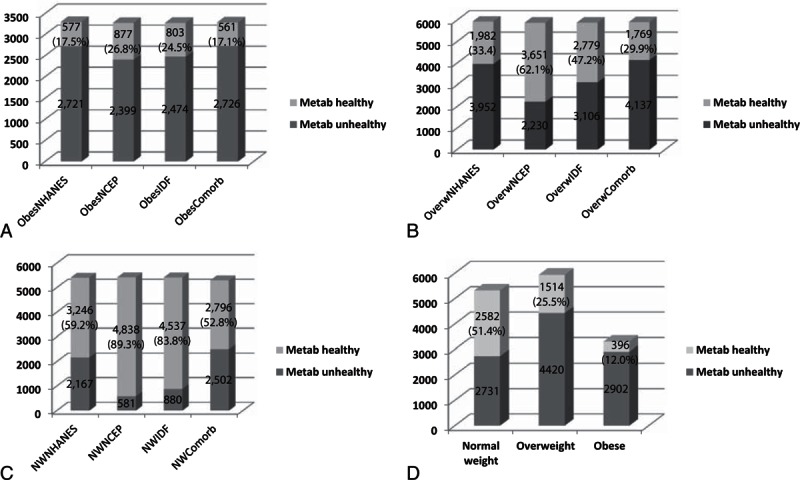
Obesity (A), overweight (B), and normal weight (C) metabolically healthy or unhealthy according to the 4 criteria, and metabolically healthy status according to the combination of all the 4 criteria (D). Comorb = comorbidities criteria, IDF = International Diabetes Federation criteria, NCEP = National Cholesterol Education Panel, NHANES = National Health Examination Surveys, MH = metabolically healthy, MUH = metabolically unhealthy.

MHS was present in 12.0% (n = 396) of the obese, 25.5% (n = 1514) of overweight, and 48.6% (n = 2582) of normal weight study participants, respectively (Table 2 and Fig. 1). The frequency of MHO was 17.5% (n = 577), 26.8% (n = 877), 24.5% (n = 803), and 17.1% (n = 561), according to NHANES, NCEP, IDF, and comorbidities criteria (Fig. 1). Agreement between all 4 criteria was strong and significant (kappa 0.73 *P* < 0.001). The agreement of MHO, overweight, and normal weight across the 4 different criteria is presented in Figure S1. Characteristics of each category are demonstrated in supplemental content (See Tables S2, S3, S5, and S5, supplemental content, which illustrates the characteristics of NHANES, NCEP, IDF, and comorbidities categories, respectively).

**Table 2:**
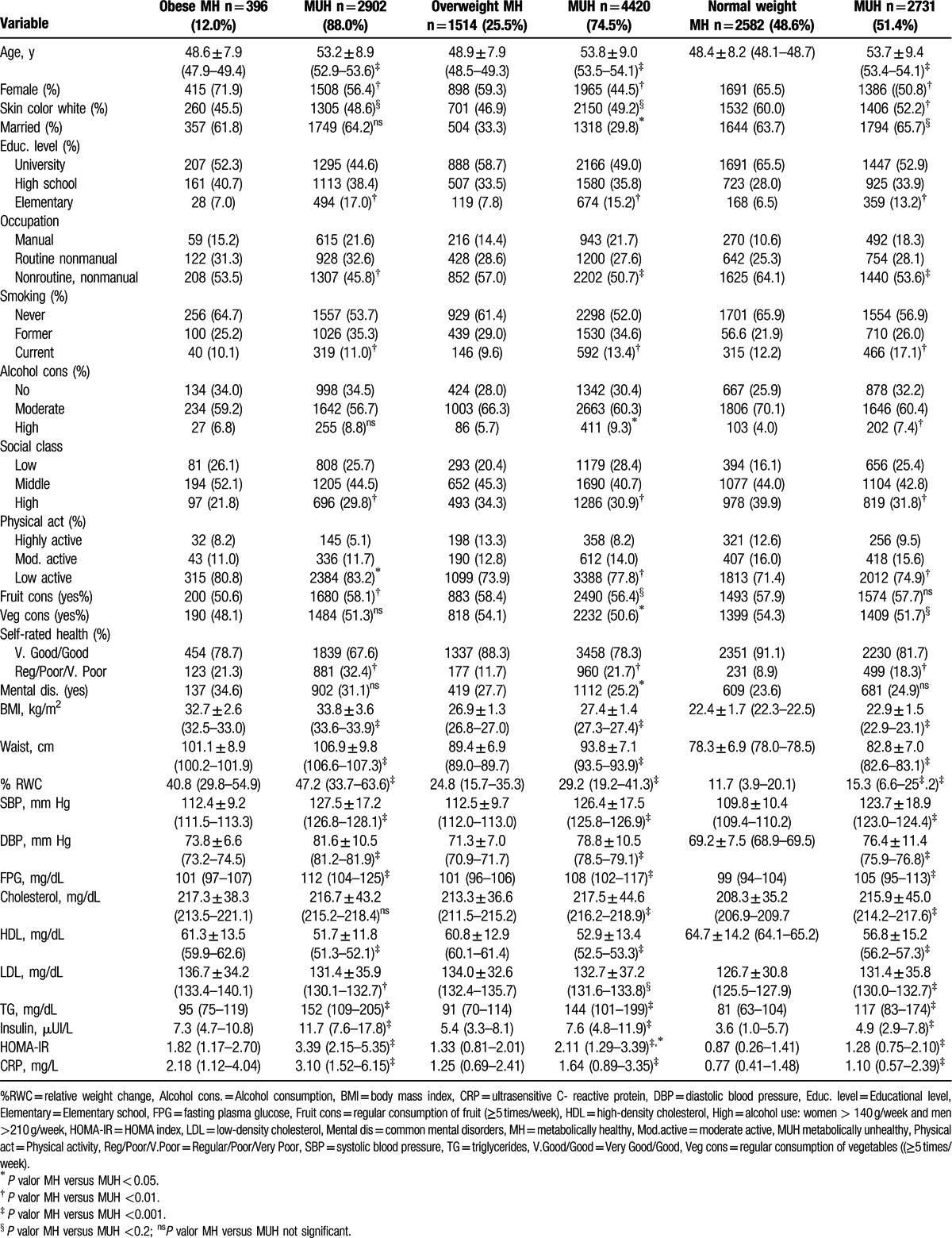
Socio-demographic, anthropometric, and laboratorial characteristics of metabolically healthy and unhealthy obese, overweight, and normal weight participants of ELSA-Brasil (baseline 2008–2010).

### 3.2 Socio-demographic and anthropometric factors associated with metabolic health status

In the univariate analysis of each of the 3 groups (normal weight, overweight, and obese), a healthy metabolic status was associated with younger age, female sex, higher educational level and social class, higher self-rated health; and healthy behaviors (no smoking and higher physical activity levels) (Table 2). Although being physically active according to WHO criteria and routine manual occupation was inversely associated with MHS in all BMI categories in univariate analysis, these associations did not remain significant after full adjustment (data not shown). In addition, lower BMI and smaller relative weight change were significantly associated with being metabolically healthy. Skin color, healthy dietary habits, alcohol consumption, and common mental disorders were not consistently associated with MHS. Premature birth did not differ between MHS across the obese (*P* = 0.74), overweight (*P* = 0.44), and normal weight (*P* = 0.66) groups. There was a strong correlation between educational level and social class (p < .001). When we analyzed the variables associated with MHS according to the 4 different criteria (NHANES, NCEP, IDF, and comorbidities), results were quite similar (See Tables S2 to S8, supplemental content, which illustrate the final logistic regression models for NHANES, NCEP, IDF, and comorbidities criteria).

In adjusted logistic models, lower age was related to MHS, so that the risk of being unhealthy increased by 6% to 7% for each additional year of life. Female sex, good self-rated health, lower BMI, and % relative weight change were associated with being metabolically healthy in each BMI category.

**Figure 2:**
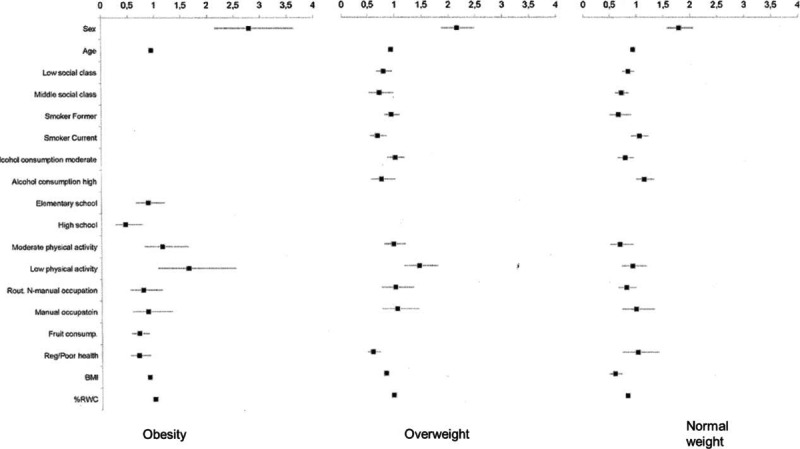
Odds ratio for factors associated with being metabolically healthy, among obese, overweight and normal weight individuals. Educational level-university degree as reference, occupation: Nonroutine, nonmanual as reference; social class: high as reference, smoking: never as reference, physical activity highly active as reference, fruit consumption: ≥5 times/week as reference, Regular/Poor/Very poor self-rated health: very good or good as reference. BMI = body mass index, Fruit consump = regular consumption of fruit (≥5 times/week), Rout.N-manual occupation = Routine nonmanual occupation, Reg./poor health = Regular/Poor/Very poor self-rated health, %RWC = relative weight change.

The odds of being metabolically healthy decreased 11% to 18% for each extra 1 kg/m^2^ of BMI, within the same BMI category, and 1% to 2% for each 1% weight increment from age 20 to the study baseline (Table 3 and Fig. 2). Lower social class was independently related to lower odds ratios (ORs) of MHS in the overweight [OR 0.69; 95% confidence interval (CI) 0.57–0.83] and normal weight groups (OR 0.65; 95% CI 0.54–0.78), but not in the obese group.

**Table 3:**
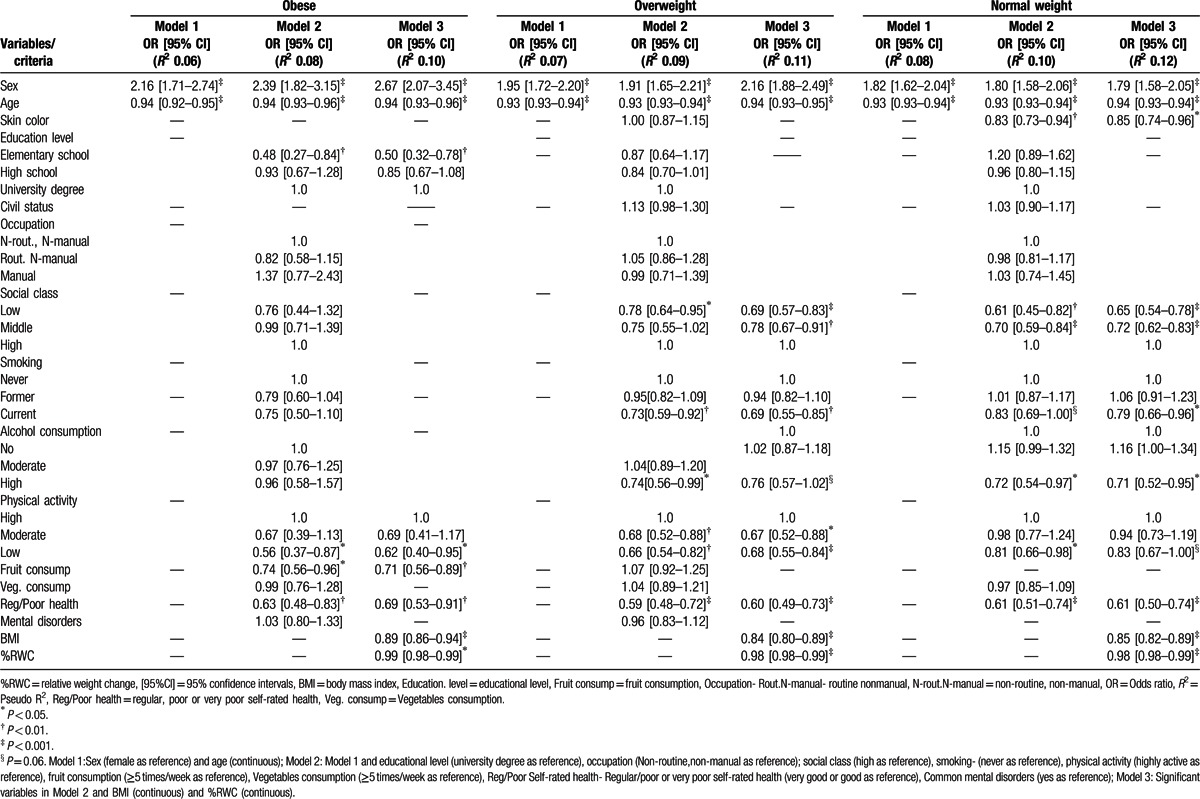
Odds ratio [95% confidence intervals] for factors associated with being metabolically healthy, among obese, overweight, and normal weight individuals.

## 4 Discussion

In this large sample of predominantly overweight and obese participants from the ELSA-Brasil cohort study, only one-third of the individuals were metabolically healthy according to all 4 MHS criteria. Systematic reviews and meta-analyses of population-based studies have identified a large variability in the prevalence of MHS among those who are obese and those of (6.0–75.0%^[19]^ and 66.1–95.9%, respectively).^[20]^ The proportion of MHS within the normal weight subgroup was lower in comparison to results in the Netherlands (97.3%),^[21]^ the United States (76.5%),^[10]^ and Spain (93.5%),^[22]^ but higher than in Finland (20.4–23.8%)^[23]^ and China (16.0%).^[24]^ This might be due not only to the more strict approach we undertook to define metabolic health^[10]^ but also to age and behavioral differences among these populations.

The proportion of having MHS across BMI categories varied according to the criterion applied, although good agreement was found in the obese category, differently from what was demonstrated by Phillips et al.^[25]^ However, agreement between the criteria was not as good within the overweight and normal weight subgroups (kappa 0.58 and 0.43, respectively). All this points out that it is difficult to compare different classifications, as stressed by other authors,^[25,26]^ and that defining metabolic health remains challenging. The term “metabolically equipoised” might be more appropriate, as the absence of cardiometabolic dysfunction can be transitory.^[6,20]^ Criteria that include biomarkers of insulin resistance (insulin, HOMA-IR) and inflammation (CRP), though theoretically more accurate, have the downside of being based on subjective cut-offs, so that each population sample will exhibit diverse values. In addition, in clinical and epidemiological settings, classifications that do not demand expensive examinations are preferable as long as they correlate well with the clinical ones. In our study, the NHANES criteria, which applied more complex biomarkers, had good agreement with the comorbidities criteria across all BMI categories (kappa 0.63, 0.72, and 0.70 for obesity, overweight, and normal weight, respectively).

MHS was associated with younger age in all BMI subgroups, as demonstrated by others.^[10,19,27]^ However, Yoo et al^[28]^ studying a younger sample (median age 37.2 years) and Martínez-Larrad et al,^[26]^ with a Spanish-Caucasian sample of adults could not confirm that. After full adjustment, female sex was associated with MHS within the BMI subgroups, as demonstrated by similar findings.^[19,29]^ The fact that women are more likely to use health services than men and, therefore, can have more opportunities for receiving preventive counseling, is one possible explanation.

Higher educational level was associated with MHO in the present study. Educational level, which is a proxy for socioeconomic and cultural status, influences living conditions and opportunities for having a healthy lifestyle. Interestingly, social class, which is strongly related to educational level, was associated with MHS only within the overweight and normal weight subgroups. A low education level and low household income link cardiovascular disease with risk factors such as smoking, hypertension, impaired glucose tolerance, diabetes mellitus, physical inactivity, and being overweight with associated metabolic disturbances.^[30]^

Another interesting point is that higher levels of self-assessed health, a valid measure of health status, increased the odds of being metabolically healthy in all BMI categories. Self-rated health has been associated with glycemic abnormalities, incident diabetes, and diabetes-related mortality in longitudinal studies.^[31]^ It is hypothesized that self-rated health reflects health problems, general physical and mental functioning, and educational levels that might be in the pathway between lifestyle behaviors and metabolic abnormalities.^[32]^

Regarding the association between MHS and lifestyle habits, we found that higher physical activity levels determined MHS in the BMI subgroups. Regular physical activity has been associated with a better profile among the components of metabolic syndrome, such as arterial pressure, lipids, and glycemia. Moreover, physical inactivity is an important risk factor for cardiovascular disease.^[14]^ Differently from ELSA-Brasil, a study that also used the IPAQ instrument could not demonstrate any association among MHS and physical activity in obese participants^[25]^, whereas in NHANES and in the International Population Study on Macro/Micronutrients and Blood Pressure (INTERMAP), MHS was related to higher physical activity levels.^[10,33]^ Another cross-sectional study that used the IPAQ questionnaire could not demonstrate any association between moderate/higher activity levels and the lipid profile of the overweight and obese participants.^[34]^ Questionnaires of physical activity cannot provide the same precision in measurement that one would achieve with objective devices. It is also noteworthy that only 5.4% of the obese participants in our population had high levels of physical activity. Despite that, as higher physical activity levels are inversely related to manual occupation, it seems that our results are really related to leisure time and transportation. We also tested physical activity according to WHO criteria, but this adjustment variable was not applied to the final logistic models. It was interesting to note that only 3495 (24.4%) of ELSA-Brasil participants are physically active according to WHO criteria. This is much lower than the Brazilian telephone-based survey, which estimates that 39.9% residents adults of 27 capitals are active, according to the same criteria.^[2]^

Interestingly, although tobacco is related to a higher risk of diabetes and hyperglycemia, as well as arterial hypertension and detrimental effects on lipids, such as lower HDL-C levels, smoking was related to a poorer metabolic profile in the present study only for the overweight and normal weight participants. Similarly, no significant relationship was found between MHS and smoking in obese individuals,^[26]^ or in participants in other BMI categories^[25]^ in other populations. Conversely, MHS was demonstrated to be inversely related to smoking by some authors.^[10,21]^ In the present study, higher alcohol consumption was associated with a metabolically unhealthy profile only in the normal weight group. On the contrary, moderate (not excessive) use of alcohol was not associated with MHS in normal weight participants, contrary to what Wildman et al^[10]^ demonstrated.

Contrary to our hypothesis, regular daily consumption of fruits and vegetables, which are surrogates of a healthy diet, was not associated with MHS. Paradoxically, regular daily consumption of fruits was associated with unhealthy metabolically status among the obese group. Similar to our findings, although having used different analyses of dietary patterns, other cross-sectional studies have not demonstrated any association between diet and MHS in cohorts.^[33–36]^ In cross-sectional studies, it is not possible to assess the influence of prior dietary counseling with previous diagnosis of obesity or comorbidities, which could explain these findings.

MHS was also associated with lower BMI levels within each BMI category. Like in the NHANES^[10]^ and in the Korean National Health and Nutrition Examination Survey,^[27]^ we found that lower waist circumference was also associated with MHS (data not shown). However, we decided to adjust only for BMI in the final logistic model, due to the easy applicability of this measure in both clinical and epidemiologic settings. Another interesting finding was the association between lower relative weight gain and MHS within each BMI category. Even in normal weight participants, the lower the weight gain during adult life, the higher the risk of being metabolically healthy. In the Nurses Health Study, women and men who gained 5.0 to 9.9 kg from 18 to 20 years had a 1.5 to 3.0 higher risk of coronary heart disease, arterial hypertension, and diabetes in comparison with who maintained their weight.^[37]^

The large sample of adults living in a middle-income country, where the impact of socio-demographic and nutritional transition as well as the burden of chronic noncommunicable diseases are not completely understood are major strengths of this study. However, our results may not be generalizable, as our population is different from the adult populations of higher income countries. The methodological rigor in data collection, the centralized analysis of the laboratory tests, the quality assurance control, and the large set of covariables are other strengths of the ELSA-Brasil cohort study. In view of the lack of a standard definition, we decided to use the combination of 4 different criteria to define metabolic status, which increased specificity. Therefore, MHS is very well-defined. Regarding study limitations, we point out the cross-sectional nature of the study and that final sample of this study excluded 3.7% (n = 560) of the original cohort, but that was necessary to better standardize the MHS criteria. Another limitation of the study is the unavailability of data on nonmetabolic chronic conditions, such as chronic obstructive respiratory disease or cancer, which might have introduced some residual confounding to the analyses. As well, ELSA-Brasil used a relatively simple method of estimating leisure time and transportation-related physical activity, assessed by the IPAQ questionnaire. However, IPAQ is a validated method according to World Health Organization. In addition, the lack of data on body composition at the baseline in ELSA-Brasil does not allow us to better assess the impact of lean or fat mass on MHS.

Baseline data of this large cohort showed that, despite differences in prevalence, MHS was associated with common characteristics at every BMI category. A follow-up of this cohort with repeated measures of socio-demographic, anthropometric, behavioral, and laboratorial characteristics may help to further improve our understanding of the determinants associated with maintaining a MHS.

## Supplementary Material

Supplemental Digital Content
