# Supplementary material for: Factors associated with metabolically healthy status in obesity, overweight, and normal weight at baseline of ELSA-Brasil
Source: Medicine (Baltimore). 2016 Jul 8;95(27):e4010. doi: 10.1097/MD.0000000000004010 (PMC5058808; doi:10.1097/MD.0000000000004010)
Supplement: Supplemental Digital Content [file medi-95-e4010-s001.pdf]

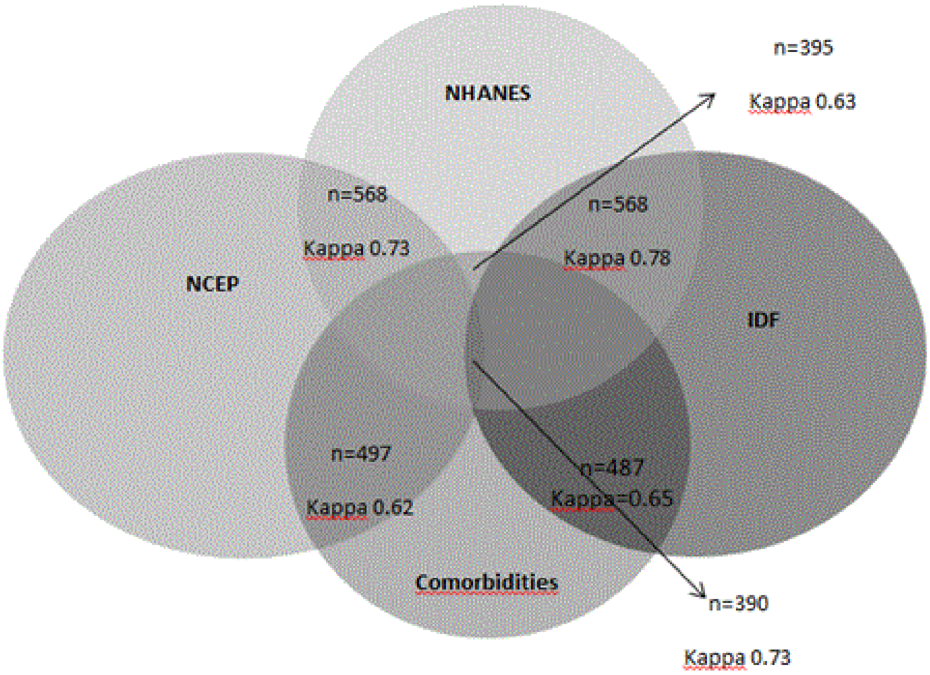

Figure S1- Agreement between criteria for metabolically healthy obesity

NHANES- National Health Examination Surveys, IDF - International Diabetes Federation, NCEP- National Cholesterol Education Panel

Table S1. Metabolic health status criteria for any body mass index category

Criteria	Alterations	MHS criteria
NHANES[10]	SBP≥130mmHg or DBP≥90mmHg or antihypertensive drugs<br>TG ≥ 1.7mmol/L or specific medication<br>HDL-C < 1.03 mmol/L (men) or specific medication<br>< 1.39 mmol/L (women) or specific medication<br>FPG ≥ 5.55 mmol/L or antidiabetic drugs<br>HOMA-IR>90th percentile (> 4.97)<br>CRP >90th percentile (> 6.25)	< 3 of the alterations
NCEP[16]	Waist circumference > 102cm (Men) or > 88 cm (Women)<br>TG ≥ 1.7mmol/L or specific medication<br>HDL-C < 1.03 mmol/L (men) or specific medication<br>< 1.29 mmol/L (women) or specific medication<br>SBP≥130mmHg or DBP≥85mmHg or arterial hypertension diagnosis<br>FPG ≥ 6.1 mmol/L or diabetes	< 3 of the alterations

	diagnosis	
IDF[17]	Waist circumference ≥ 90cm (Men) or ≥ 80 cm (Women)*<br>plus<br>TG ≥ 1.7mmol/L or specific medication<br>HDL-C < 1.03 mmol/L (men) or specific medication<br>< 1.29 mmol/L (women) or specific medication<br>SBP≥130mmHg or DBP≥85mmHg or arterial hypertension diagnosis<br>FPG ≥ 5.6 mmol/L or diabetes diagnosis	Waist circunference<br>+<br>< 2 alterations
Comorbidities	Diabetes<br>Arterial hypertension<br>Dyslipidemia	No alteration

MHS- Metabolic health status; NHANES- National Health Examination Surveys; NCEP- National Cholesterol Education Panel; IDF- International Diabetes Federation; SBP- Systolic blood pressure; DBP- diastolic blood pressure; TG- Triglycerides; HDL-C- High density cholesterol; FPG- fasting plasma glucose; HOMA-IR - Homeostasis model assessment; CRP- ultrasensitive C-reactive protein; * central obesity to south and central Americans, according to IDF

Table S2 – Socio-demographic, anthropometric and laboratorial characteristics of metabolically healthy and unhealthy obese, overweight and normal weight participants according to criteria National Health Examination Surveys [10]

**Variable**	**Obesity**		**Overweight**		**Normal weight**	
	**MH**	**MUH**	**MH**	**MUH**	**MH**	**MUH**
	**n=577 (17.5%)**	**n=2,721(82.5%)**	**n=1,982 (33.4%)**	**n=3,952(66.6%)**	**n=3,246(59.2%)**	**n=2,167(40.8%)**
**Age** (y)	49.2 ± 8.2 (48.5-49.8)	53.4± 8.8 (53.1-53.8) ***	49.2±8.1 (48.8-49.5)	54.2±8.9 (53.9-54.5)***	48.8±8.3 (48.3-49.1)	54.6±9.3 (54.2-55.0)***
**Female** (%)	415(71.9)	1,508(55.4)***	1,182(56.9)	1,681(42.5)***	2,057(65.4)	1,020(47.1)***
**Skin color white** (%)	260(45.5)	1,305(48.6)$^{\delta}$	1,014(51.8)	1,999(51.2)^{ns}	1,805(58.0)	1,133(53.0)***
**Married** (%)	357 (61.9)	1,749(64.3)^{ns}	1,318(66.5)	2,794(70.7)**	2,047(63.0)	1,463(66.5)***
**Educ. level (%)**						
University	291(50.4)	1,211(44.5)	1,114(56.2)	1,940(49.1)	2,010(63.9)	1,128(52.1)
High school	240(41.6)	1,034(38.0)	693(35.0)	1,394(35.3)	904(28.7)	744(34.3)
Elementary school	46(8.0)	476(17.5)***	175(8.8)	618(15.6)***	232(7.4)	295(13.6)***
**Occupation**						
Non-rout.,non-manual	285(50.3)	1,230(46.0)	1,069(54.7)	1,985(51.1)	1,930(62.5)	1,135(53.2)
Routine manual	196(34.5)	854(32.0)	571(29.2)	1,057(27.2)	808(26.1)	588(27.6)
Manual	86(15.2)	588(22.0)**	314(16.1)	845(21.7)***	353(11.4)	409(192)***
**Smoking(%)**						
Never	381(66.0)	1,432(52.6)	1,203(60.7)	2,024(51.2)	2,050(65.1)	1,205(55.6)
Former	142(24.6)	984(36.2)	559(28.2)	1,410(35.7)	681(21.7)	595(27.5)
Current	54(9.4)	305(11.2)***	220(11.1)	518(13.1)***	415(13.2)	366(16.9)***
**Cons.Alcohol** (%)						
No	204(35.3)	928(34.2)	571(28.8)	1,195(30.3)	853(27.2)	692(32.0)
Moderate	339(59.0)	1,537(56.6)	1,292(65.3)	2,374(60.1)	2,149(68.5)	1,303(60.2)
High	33(5.7)	249(9.2)**	117(5.9)	380(9.6)***	136(4.3)	169(7.8)***
**Physical act(%)**						
Highly active	42(7.4)	135(5.0)	247(12.7)	309(7.9)	373(12.0)	204(9.6)
Moderate active	65(11.4)	314(11.7)	248(12.7)	554(14.2)	472(15.2)	353(16.6)
Low active	464(81.3)	2,235(83.3)$^{\delta}$	1,458(74.8)	3,029(77.9)***	2,252(72.8)	1,573(73.8)**
**Cons.fruit(yes%)**	312(54.2)	1,568(57.8)$^{\delta}$	1,147(57.9)	2,226(56.4)^{ns}	1,799(57.3)	1,268(58.6)^{ns}
**Cons.vege(yes%)**	292(50.7)	1,382(50.9)^{ns}	1,053(53.2)	1,997(50.6)$^{\delta}$	1,694(53.9)	1,114(51.5)$^{\delta}$
**Self-rated health(%)**						
Very good/Good	454(78.7)	1,839(67.6)	1,734(87.5)	3,061(77.5)	2,816(89.5)	1,765(81.5)

Reg/Poor/VeryPoor	123(21.3)	881(32.4)***	248(12.5)	889(22.5)***	329(10.5)	401(18.5)***
**Mental dis.(yes)**	198(34.3)	841(30.9)$^{\delta}$	554(27.9)	977(24.7)**	760(24.2)	530(24.5)^{ns}
**Social class(%)**						
Low	124(22.8)	765(30.1)	605(32.3)	1,174(31.6)	512(17.2)	538(26.3)
Middle	281(51.9)	1,118(44.1)	854(45.6)	1,488(40.0)	1,335(44.8)	846(41.3)
High	137(25.3)	656(25.8)***	414(22.1)	1,058(28.4)***	1,134(38.0)	663(32.4)***
**Premature birth**	29(5.1)	126(4.8)^{ns}	88(4.5)	200(5.2)^{ns}	185(6.0)	113(5.3)^{ns}
**BMI (kg/m^2)**	32.8 ± 2.6 (32.5-32.9)	33.8 ± 3.6 (33.7-33.9)***	26.9 ± 1.4 (26.9-27.1)	27.4 ± 1.4 (27.4-27.5)***	22.4 ± 1.6 (22.4-22.5)	23.1 ± 1.5 (23.0-23.1)***
**Waist (cm)**	101.3 ± 9.1 (100.6-102.1)	107.3 ± 9.8 (106.9-107.7)***	89.6 ± 7.1 (89.3-89.9)	94.2 ± 6.9 (93.9-94.4)***	78.6 ± 6.9 (78.4-78.8)	83.3 ±6.9 (83.2-83.8)***
**% RWC**	40.8(29.2-54.7)	47.5(33.9-64.0)***	25.6(16.0-35.9)	29.4(19.2-41.6)***	11.8(3.6-20.3)	15.4(6.4-25.8)***
**SBP (mmHg)**	114.4± 11.9 (113.4-115.3)	128.1±17.2 (127.4-128.7)***	114.3±11.9 (113.8-114.8)	127.1±17.5 (126.6-127.7)***	111.5±12.6 (111.0-111.9)	125.0±18.9 (124.2-125.8)***
**DBP (mmHg)**	74.8 ±7.7 (74.2-75.5)	81.9 ± 10.5 (81.5-82.3)***	72.6 ± 8.2 (72.2-72.9)	79.1± 10.5 (78.7-79.4)***	70.1±8.6 (69.8-70.4)	77.0 ± 11.4 (76.5-77.5)***
**FPG (mg/dL)**	99(95-106)	112(105-126)***	99(95-106)	109(103-118)***	98(94-104)	107(102-114)***
**Total Cholesterol** (mg/dL)	217.4±37.9 (214.3-220.5)	216.7±43.6 (215.1-218.4)^{ns}	213.6±38.0 (211.9-215.3)	217.9±44.8 (216.5-219.3)***	208.4±36.8 (207.2-209.7)	217.3±45.1 (215.4-219.1)***
**HDL-C (mg/dL)**	59.6±13.2 (58.6-60.7)	51.4±11.8 (50.9-51.9)***	59.8±13.6 (59.2-60.4)	52.5±13.2 (52.1-52.9)***	63.9±14.9 (63.4-64.4)	56.2±14.9 (55.6-56.9)***
**LDL (mg/dL)**	136.5 ±32.6 (133.9-139.2)	131.1 ±36.2 (129.7-132.5)**	133.9 ±33.0 (132.5-135.4)	132.6±37.5 (131.5-133.8)$^{\delta}$	126.8±31.6 (125.7-127.8)	131.8 ±35.8 (130.3-133.3)***
**TG (mg/dL)**	99(78-127)	155(112-209)***	94(72-119)	151(105-204)***	82(64-107)	125(88-180)***
**Fasting insulin (µUI/L)**	7.6(4.9-11.0)	12.1(7.8-18.3)***	5.4(3.3-8.4)	7.9(5.0-12.2)***	3.7(1.0-5.8)	5.1(2.9-8.0)***
**HOMA-IR**	1.9(1.2-2.8)	3.5(2.3-5.5)***	1.4(0.8-2.1)	2.2(1.4-3.5)***	0.9(0.3-1.4)	1.4(0.8-2.2)***
**CRP (mg/L)**	2.3(1.2-4.1)	3.2(1.5-6.4)***	1.3(0.7-2.5)	1.7(0.9-3.4)***	0.8(0.4-1.5)	1.2(0.6-2.6)***

MH- metabolically healthy; **MUH** metabolically unhealthy; **Educ. level-** Educational level**;** Non-rout.,non-manual- Non-routine, non-manual; **Undergrad. or more-** undergraduate or more; **Cons. alcohol-** Consumption of alcohol; High- alcohol use > 140g/week/women and >210g/week/men**; Physical act –** Physical activity**; Cons.fruit-** regular consumption of fruit ((≥5 times/week); **Cons.vege-** regular consumption of vegetables((≥5 times/week); **Reg/Poor/VeryPoor-** Regular/Poor/Very Poor; **Mental dis.-** common mental disorders; **BMI**- body mass index;**%RWC-** relative weight change**; SBP-** systolic blood pressure**; DBP-** diastolic blood pressure; **FPG**- fasting plasma glucose; **HDL-C**- high density cholesterol; **LDL**- low density cholesterol;**TG**- triglycerides; **HOMA-IR** - HOMA index ; **CRP**- C reactive protein

*p valor MH versus MUH <.05; ** p valor MH versus MUH <.01; *** p valor MH versus MUH <.001; $^{\delta}$ p valor MH versus MUH <.2; ^{ns} p valor MH versus MUH not significant

Table S3 – Socio-demographic, anthropometric and laboratorial characteristics of metabolically healthy and unhealthy obese, overweight and normal weight participants according to criteria National Cholesterol Education Panel [16]

**Variable**	**Obesity**		**Overweight**		**Normal Weight**	
	**MH**	**MUH**	**MH**	**MUH**	**MH**	**MUH**
	**877 (26.8%)**	**2,399 (73.2%)**	**3,651 (62.1%)**	**2,230 (37.9%)**	**4.838 (89.3%)**	**581 (10.7%)**
**Age** (y)	49.9±8.5 (49.4-50.6)	53.7±8.8 (53.3-54.0)***	51.0±8.7 (50.71-51.28)	55.1±8.9 (54.70-55.40)***	50.6±9.1 (50.31-50.83)	55.5±8.9 (54.76-56.22)***
**Female** (%)	599(63.3)	1,311(54.6)***	1,764(48.3)	1,076(48.3)^{ns}	2,794(59.4)	265(45.7)***
**Skin color white** (%)	406(46.8)	1,148(48.5)^{ns}	1,877(52.0)	1,111(50.4)^{ns}	2,650(57.0)	284(49.8)***
**Married** (%)	547(62.4)	1,545(64.4)^{ns}	2,540(69.6)	1,532(68.7)^{ns}	3,039(64.7)	379(65.3)^{ns}
**Education.level(%)**						
Undergrad. or more	437(49.8)	1,056(44.0)	1,978(54.2)	1,058(47.4)	2856(60.7)	268(46.2)
High school	356(40.6)	911(38.0)	1,251(34.3)	809(36.3)	1,420(30.2)	217(37.4)
Elementary school	84(9.6)	432(18.0)***	422(11.6)	363(16.3)***	426(9.1)	95(16.4)***
**Occupation**						
Non-rout.,non-manual	441(51.1)	1,066(45.3)	1,946(54.2)	1,085(49.4)	2,773(60.1)	278(48.2)
Routine non-manual	276(31.9)	764(32.5)	982(27.3)	627(28.6)	1,202(26.0)	185(32.1)
Manual	146(17.0)	524(22.2)**	665(18.5)	483(22.0)**	642(13.9)	114(19.7)***
**Smoking (%)**						
Never	560(63.8)	1,244(51.9)	2,117(58.0)	1,088(48.8)	2,927(62.3)	310(53.5)
Former	232(26.5)	883(36.8)	1,114(30.5)	838(37.6)	1,113(23.6)	159(27.5)
Current	85(9.7)	272(11.3)***	420(11.5)	304(13.6)***	662(14.1)	110(19.0)***
**Cons. Alcohol**						
No	283(32.3)	840(35.1)	1,036(28.4)	710(31.9)	1,339(28.5)	196(33.8)
Moderate	541(61.7)	1,326(55.4)	2,353(64.5)	1,288(57.8)	3,107(66.2)	332(57.2)
High	53(6.0)	228(9.5)***	259(7.1)	231(10.3)***	249(5.3)	52(9.0)***
**Physical act(%)**						
Highly active	69(7.9)	105(4.4)	423(11.7)	129(5.8)	533(11.5)	43(7.7)
Moderate active	97(11.2)	282(12.0)	491(13.7)	303(13.8)	741(16.0)	80(14.0)
Low active	704(80.9)	1,978(83.6)***	2,680(74.6)	1,767(80.4)***	3,360(72.5)	443(78.3)**
**Cons. fruit(yes)**	485(55.3%)	1,385(57.9)$^{\delta}$	2,061(56.5%)	1,277(57.3%)^{ns}	2,731(58.2)	318(54.8)$^{\delta}$
**Cons. veget.(yes)**	455(51.8%)	1,210(50.6%)^{ns}	1,910(52.4)	1,115(50.0%)$^{\delta}$	2,505(53.4)	288(49.7)$^{\delta}$
**Self-rated health(%)**						
Very good/Good	684(78.0)	1,591(66.3)	3,117(85.4)	1,638(73.5)	4,107(87.4)	454(78.3)

Reg/Poor/VeryPoor	193(22.0)	807(33.7)***	534(14.6)	590(26.5)***	593(12.6)	126(21.7)***
**Mental dis.(yes)**	276(31.4%)	755(31.5%)[ns]	925(25.3%)	593(26.6%)[ns]	1,119(23.8)	163(28.1)**
**Class Social**<br>Low<br>Middle<br>High	 210(25.4)<br>398(48.1)<br>218(26.4)	 674(30.2)<br>990(44.3)<br>569(25.5)*	 855(24.8)<br>1,450(42.0)<br>1,144(33.2)	 603(28.8)<br>866(41.4)<br>624(29.8)***	 880(19.7)<br>1,932(43.4)<br>1,644(36.9)	 162(29.7)<br>236(43.3)<br>147(27.0)***
**Premature birth(yes%)**	45(5.2)	108(4.6)[ns]	165(4.6)	116(5.3)[ns]	260(5.6)	37(6.5)[ns]
**BMI (kg/m^2)**	32.9 ± 3.0 (32.8-33.2)	33.9 ± 3.6 (33.8-34.0)***	27.0 ± 1.3 (26.9-27.0)	27.7 ± 1.4 (27.6-27.8)***	22.6 ± 1.6 (22.6-22.7)	23.4 ± 1.3 (23.3-23.5)***
**WC (cm)**	101.8 ± 9.5 (101.2-102.4)	107.9 ± 9.6 (107.5-108.3)***	90.7± 6.9 (90.5-90.9)	95.8± 6.8 (95.5-96.1)***	79.9 ± 7.1 (79.70-80.10)	86.4 ± 6.3 (85.90-86.90)***
**% RWC**	42.7(30.7-57.3)	47.8(34.0-64.1)***	27.4±16.8(26.9-27.9)	34.0 ±18.9(33.2-34.8)***	12.9(4.8-22.1)	18.5(9.8-29.4)***
**SBP (mmHg)**	116.1±12.7 (115.2-116.9)	129.2±17.3 (128.5-129.9)***	118.6±14.8 (118.2-119.1)	129.8±18.1(129.1-130.6)***	115.2±15.7(114.8-115.7)	130.2±19.1(128.6-131.7)***
**DBP (mmHg)**	75.6 ±8.1 (75.1-76.1)	82.5 ±10.6 (82.1-82.9)***	74.8±9.3 (74.5-75.1)	80.3±10.9 (79.9-80.8)***	72.1±9.8 (71.8-72.3)	79.8 ±11.6 (78.9-80.8)***
**FPG (mg/dL)**	101(96-109)	113(106-128)***	103(97-110)	111(105-122)***	101(95-107)	110(104-119)***
**Total Cholesterol** (mg/dL)	215.0±37.0 (212.6-217.5)	217.5±44.5 (215.7-219.3)$^{\delta}$	214.0±40.1 (212.7-215.3)	220.5±46.4 (218.6-222.4)***	210.7±38.9 (209.6-211.8)	224.6±51.3 (220.5-228.8)***
**HDL-C (mg/dL)**	58.7±12.6 (57.9-59.5)	50.7±11.6 (50.2-51.2)***	57.5±13.7 (57.1-57.9)	50.7±12.8 (50.2-51.2)***	61.9±14.9 (61.6-62.4)	49.6±12.6 (48.5-50.6)***
**LDL (mg/dL)**	134.3±32.5 (132.1-136.4)	131.3±36.8 (129.8-132.8)*	133.3±34.0 (132.2-134.4)	132.7±39.2 (131.1-134.3)[ns]	128.5±32.8 (127.6-129.4)	134.1±38.1 (130.9-137.2)***
**TG (mg/dL)**	103(81-128)	162(118-217)***	104(78-135)	173(130-226)***	90(68-119)	181.5(153-241)***
**Fasting insulin (µUI/L)**	8.7(5.5-12.8)	12.1(8.0-18.4)***	6.1(3.8-9.3)	8.9(5.5-13.7)***	4.1(2.1-6.3)	6.1(3.8-9.5)***
**HOMA-IR**	2.22(1.37-3.39)	3.56(2.31-5.61)***	1.56(0.96-2.46)	2.5(1.59-4.07)***	1.0(0.5-1.6)	1.8(1.0-2.9)***
**CRP (mg/L)**	2.62(1.24-5.22)	3.10(1.53-5.88)*	1.35(0.74-2.76)	1.86(1.00-3.67)***	0.89(0.46-1.79)	1.43(0.72-2.70)***

MH- metabolically healthy; **MUH** metabolically unhealthy; **Education. Level-** educational level; **Non-rout.,non-manual**- Non-routine, non-manual;**Undergrad. or more**-undergraduate or more; **Cons. alcohol-** Consumption of alcohol; High- alcohol use > 140g/week/women and >210g/week/men**; Physical act -** Physical activity **; Cons.fruit-** regular consumption of fruit ((≥5 times/week); **Cons.vege-** regular consumption of vegetables((≥5 times/week); **Reg/Poor/VeryPoor –** Regular/Poor/Very poor**; Mental dis.-** common mental disorders; **BMI**- body mass index;**%RWC-** relative weight change**; SBP-** systolic blood pressure**; DBP-** diastolic blood pressure; **FPG-** fasting plasma

glucose; **HDL-C**- high density cholesterol; **LDL**- low density cholesterol;**TG**- triglycerides; **HOMA-IR** - HOMA index ; **CRP**- C reactive protein. *p valor MH versus MUH <.05; ** p valor MH versus MUH <.01; *** p valor MH versus MUH <.001; $^{\delta}$ p valor MH versus MUH <.2; ^{ns} p valor MH versus MUH not significant

Table S4 – Socio-demographic, anthropometric and laboratorial characteristics of metabolically healthy and unhealthy obese, overweight and normal weight participants according to criteria International Diabetes Federation [17]

Variable	Obesity		Overweight		Normal weight	
	**MH 803(24.5%)**	**MUH 2,474(75.5%)**	**MH 2,779(47.2%)**	**MUH 3,106(52.8%)**	**MH 4,537 (83.8%)**	**MUH 880 (17.2%)**
**Age** (y)	49.9±8.6 (49.3-50.5)	53.6±8.8 (53.3-53.9)***	50.3±8.5 (49.9-50.6)	54.6±8.9 (54.2-54.9)	50.3±9.0 (50.0-50.5)	55.2±9.0 (54.6-55.8)
**Female** (%)	584(72.7)	1,326(53.6)***	1,464(52.7)	1,375(44.3)***	2,554(58.0)	503(57.2)^{ns}
**Skin color white** (%)	376(47.3)	1,178(48.3)^{ns}	1,438(52.4)	1,553(50.5)$^{\delta}$	2,490(57.2)	441(50.9)**
**Married** (%)	489(60.9)	1,604(64.8)*	1,898(68.3)	2,177(70.1)$^{\delta}$	2,843(64.6)	574(65.3)^{ns}
**Educational level (%)**						
University	409(50.9)	1.084(43.8)	1,544(55.6)	1,492(48.0)	2,682(60.9)	440(50.1)
High school	326(40.6)	942(38.1)	946(34.0)	1.118(36.0)	1,329(30.2)	307(34.9)
Elementary school	68(8.5)	448(18.1)*	289(10.4)	496(16.0)***	390(8.9)	132(15.0)***
**Occupation**						
Non-rout,non-manual	410(51.8)	1,097(45.2)	1,499(54.8)	1,533(50.2)	2,613(60.5)	436(49.8)
Routine non-manual	261(33.0)	779(32.1)	765(27.9)	846(27.7)	1,099(25.5)	288(32.9)
Manual	120(15.2)	551(22.7)***	474(17.3)	675(22.1)***	606(14.0)	151(17.3)***
**Smoking (%)**						
Never	516(64.2)	1,288(52.1)	1,625(58.5)	1,577(50.8)	2,745(62.4)	488(55.6)
Former	211(26.3)	905(36.6)	838(30.1)	1,117(35.9)	1,046(23.8)	225(25.6)
Current	76(9.5)	281(11.3)***	316(11.4)	412(13.3)***	610(13.8)	165(18.8)***
**Alcohol use**						
No	267(33.2)	856(34.7)	788(28.4)	957(30.8)	1,229(28.0)	301(34.3)
Moderate	492(61.3)	1,376(55.7)	1,814(65.4)	1,829(58.9)	2,937(66.8)	503(57.3)
High	44(5.5)	237(9.6)***	174(6.2)	319(10.3)***	228(5.2)	74(8.4)***
**Physical act(%)**						
Highly active	62(7.8)	113(4.6)	349(12.8)	205(6.7)	511(11.8)	65(7.6)
Moderate active	89(11.2)	290(11.9)	361(13.2)	434(14.2)	692(15.9)	129(15.0)
Low active	645(81.0)	2,037(83.5)**	2,023(74.0)	2,425(79.1)***	3,137(72.3)	665(77.4)**
**Cons. fruit(yes)**	457(56.9)	1,414(57.3)^{ns}	1,586(57.2)	1,756(56.6)^{ns}	2,537(57.8)	509(57.9)^{ns}
**Cons. veget.(yes)**	420(52.3)	1,245(50.5)^{ns}	1,469(52.9)	1,557(50,2)*	2,334(53.1)	457(52.1)^{ns}
**Self-rated health(%)**						
Very good/Good	625(77.8)	1,651(66.8)	2,416(86.9)	2,342(75.5)	3,865(87.9)	695(79.1)

Reg/Poor/Very Poor	178(22.2)	822(33.2)***	363(13.1)	762(24.5)***	534(12.1)	184(20.9)***
**Mental dis (yes)**	261(32.5)	770(31.2)[ns]	721(25.9)	797(25.7)[ns]	1,033(23.5)	249(28.3)**
**Social class**						
Low	182(24.0)	703(30.5)	613(23.4)	846(28.9)	814(19.5)	228(27.4)
Middle	371(49.0)	1,017(44.2)	1,133(43.2)	1,187(40.6)	1,802(43.2)	366(43.9)
High	204(27.0)	583(25.3)**	874(33.4)	893(35.5)***	1,552(37.3)	238(28.6)***
**Premature birth**	42(5.3)	111(4.6)[ns]	118(4.3)	163(5.4)[δ]	245(5.7)	52(6.1)[ns]
**BMI (kg/m^2)**	33.1 ± 3.1 (32.9-33.3)	33.8 ± 3.6 (33.7-33.9)***	26.9 ± 1.3 (26.9-27.0)	27.6 ± 1.4 (27.5-27.6)***	22.5 ± 1.6 (22.5-22.6)	23.5 ± 1.2 (23.5-23.6)***
**WC (cm)**	102.3 ±9.7 (101.6-102.9)	107.5 ±9.7 (107.2-107.9)***	89.9 ±6.9 (89.6-90.1)	95.1±6.8 (94.8-95.3)***	79.5 ±7.0 (79.3-79.7)	86.2±6.1 (85.8-86.6)***
**% RWC**	43.5(31.2-57.8)	47.3(33.7-63.9)***	25.1(15.8-36.1)	30.4(20.4-42.7)***	12.3(4.3-21.3)	19.9(10.8-29.7)***
**SBP (mmHg)**	114.9±11.6 (114.1-115.7)	129.2±17.3 (128.5-129.9)***	116.4 ±13.2 (115.9-116.9)	128.7±17.9 (128.1-129.3)***	114.7±15.4 (114.3-115.2)	127.9±19.1 (126.6-129.2)***
**DBP (mmHg)**	74.9± 7.5 (74.5-75.5)	82.5±10.6 (82.1-82.9)***	73.6 ±8.7 (73.3-73.9)	79.9± 10.7 (79.5-80.3)***	71.8 ±9.7 (71.5-72.1)	78.8±11.3 (78.0-79.5)***
**FPG (mg/dL)**	100(96-109)	113(106-128)***	101(96-108)	110(104-120)***	100(95-107)	108(103-118)***
**Total Cholesterol** (mg/dL)	215.2±37.2 (212.6-217.8)	217.3±44.2 (215.6-219.1)[δ]	213.1±39.1 (211.6-214.5)	219.5±45.5 (217.9-221.1)***	209.8±38.5 (208.7-210.9)	224.4±48.4 (221.2-227.6)***
**HDL-C (mg/dL)**	59.3±12.6 (58.5-60.2)	50.7±11.6 (50.3-51.2)***	58.8±13.9 (58.3-59.3)	51.5±12.7 (51.0-51.9)***	62.2±15.0 (61.7-62.6)	52.8±13.7 (51.9-53.7)***
**LDL (mg/dL)**	134.5±32.6 (132.2-136.7)	131.3±36.7 (129.9-132.8)*	132.8±33.8 (131.5-134.1)	133.3±37.9 (131.9-134.6)[ns]	127.8±32.5 (126.8-128.7)	135.7±37.3 (133.3-138.2)***
**TG (mg/dL)**	103(80-127)	161(117-216)***	98(75-126)	162(115-214)***	88(68-117)	164(114-214)***
**Fasting insulin (μUI/L)**	8.6(5.5-12.7)	12.1(7.9-18.3)***	5.7(3.6-8.8)	8.4(5.3-12.9)***	3.9(2.1-6.2)	5.9(3.7-9.0)***
**HOMA-IR**	2.2(1.3-3.3)	3.5(2.3-5.6)***	1.5(0.9-2.3)	2.4(1.5-3.8)***	0.9(0.5-1.5)	1.6(1.0-2.6)
**CRP (mg/L)**	2.7(1.3-5.3)	3.0(1.5-5.8)*	1.3(0.7-2.7)	1.8(0.9-3.5)***	0.9(0.5-1.7)	1.4(0.7-2.7)***

MH- metabolically healthy; **MUH** metabolically unhealthy; **University**- university or more; **Non-rout,non-manual**- Non-routine, non-manual; **Cons. alcohol-** Consumption of alcohol; High- alcohol use > 140g/week/women and >210g/week/men**; Physical act –** Physical activity**; Cons.fruit-** regular consumption of fruit ((≥5 times/week); **Cons.veget-** regular consumption of vegetables((≥5 times/week); **Reg/Poor/VeryPoor-** Regular/Poor/Very Poor; **Mental dis.-** common mental disorders; **BMI**- body mass index;**%RWC-** relative weight change**; SBP-** systolic blood pressure**; DBP-** diastolic blood pressure; **FPG**- fasting plasma glucose; **HDL-C**- high density cholesterol; **LDL**- low density cholesterol;**TG**- triglycerides; **HOMA-IR** - HOMA index ; **CRP**- C reactive protein. *p valor MH versus MUH <.05; ** p valor MH versus MUH <.01; *** p valor MH versus MUH <.001; [δ] p valor MH versus MUH <.2; [ns] p valor MH versus MUH not significant

Table S5 – Socio-demographic, anthropometric and laboratorial characteristics of metabolically healthy and unhealthy obese, overweight and normal weight participants according to criteria comorbidities

Variable	Obesity		Overweight		Normal weight	
	MH 561(17.1%)	MUH 2,726(82.9%)	MH 1,769(29.9%)	MUH 4,137(70.1%)	MH 2,796(52.8%)	MUH 2,502(47.2%)
**Age** (y)	49.5±8.0 (48.9-50.2)	53.3±8.9 (52.9-53.7)***	49.4±8.1 (48.9-49.7)	53.9±9.0 (53.6-54.2)***	48.7±8.3 (48.4-49.0)	53.9±9.4 (53.5-54.2)***
**Female** (%)	400(71.3)	1,516(55.6)***	1,014(57.3)	1,838(44.4)***	1,794(64.2)	1,274(50.9)***
**Skin color white** (%)	261(47.1)	1,2973(48.2)^{ns}	932(53.5)	2,061(50.3*	1,642(59.4)	1,281(51.9)***
**Married** (%)	337(60.1)	1762(64.6)*	1,180(66.7)	2,913(70.4)**	1,778(64.0)	1,649(66.0)$^{\delta}$
**Educational level(%)**						
University	291(51.9)	1,203(44.1)	1,017(57.5)	2,015(48.7)	1,821(65.1)	1,306(52.2)
High school	220(39.2)	1,053(38.6)	596(33.7)	1,486(35.9)	784(28.0)	860(34.4)
Elementary school	50(8.9)	470(17.3)***	156(8.8)	636(15.4)***	191(6.8)	336(13.4)***
**Occupation**						
Non-Rout, non-manual	291(52.7)	1,214(45.4)	980(56.0)	2,052(50.5)	1,749(63.7)	1,303(53.0)
Routine manual	179(32.4)	870(32.5)	499(28.5)	1,126(27.7)	691(25.1)	703(28.6)
Manual	82(14.9)	592(22.1)***	271(15.5)	886(21.8)***	309(11.2)	453(18.4)***
**Smoking (%)**						
Never	356(63.5)	1,451(53.2)	1,075(60.8)	2,140(51.7)	1,826(65.3)	1,420(56.8)
Former	145(25.9)	976(35.8)	522(29.5)	1,433(34.6)	624(22.3)	647(25.9)
Current	60(10.7)	299(11.0)***	172(9.7)	564(13.7)***	346(12.4)	434(17.4)***
**Cons Alcohol**						
No	175(31.2)	956(35.2)	500(28.3)	1,260(30.5)	729(26.1)	814(32.6)
Moderate	350(62.4)	1,517(55.8)	1,154(65.3)	2,493(60.3)	1.949(69.9)	1,490(59.7)
High	36(6.4)	246(9.0)**	114(6.4)	380(9.2)***	112(4.0)	193(7.7)***
**Physical act (%)**						
Highly active	45(8.1)	129(4.8)	218(12.6)	334(8.2)	343(12.5)	232(9.4)
Moderate active	55(9.9)	321(12.0)	219(12.6)	578(14.2)	444(16.1)	378(15.4)
Low active	453(82.0)	2,241(83.2**)	1,299(74.8)	3,169(77.6)***	1,966(71.4)	1,851(75.2)**
**Cons. fruit(yes)**	297(52.9)	1,577(58.0)*	1,019(57.7)	2,336(56.7)^{ns}	1,616(57.9)	1,438(57.6)^{ns}
**Cons.veget.(yes)**	289(51.5)	1,381(50.8)^{ns}	952(53.9)	2,084(50.5)*	1,505(53.9)	1,293(51.8)$^{\delta}$
**Self-rated health**						
Very good/Good	442(78.8%)	1,841(67.6%)	1,560(88.2%)	3,210(77.6%)	2,543(91.0)	2,025(81.0)

Reg/Poor/Very Poor	119(21.2%)	884(32.4%)***	209(11.8%)	925(22.4%)***	253(9.0)	475(19.0)***
**Mental dis(yes)**	187(33.3)	851(31.3)^{ns}	488(27.6)	1,032(25.0)*	662(23.7)	626(25.0)^{ns}
**Social Class**<br>Low<br>Middle<br>High	 113(21.5)<br>271(51.4)<br>143(27.1)	 774(30.4)<br>1,126(44.3)<br>643(25.3)***	 358(21.3)<br>753(44.8)<br>571(33.9)	 1,111(28.6)<br>1,583(40.7)<br>1,191(30.7)***	 441(16.6)<br>1,161(43.7)<br>1,056(39.7)	 607(25.8)<br>1,016(43.1)<br>733(31.1)***
**Premature birth (yes%)**	29(5.2)	125(4.7)***	81(4.7)	206(5.1)^{ns}	159(5.8)	139(5.7)^{ns}
**BMI (kg/m^2)**	33.1 ± 3.2 (32.9-33.4)	33.8 ± 3.5 (33.6-33.9)***	26.9 ± 1.4 (26.9-27.1)	27.4 ± 1.4 (27.3-27.4)***	22.4 ± 1.7 (22.3-22.5)	23.0 ± 1.5 (22.9-23.1)***
**WC (cm)**	102.2 ± 9.5 (101.4-102.9)	107.1 ± 9.8 (106.7-107.5)***	89.8±7.1 (89.5-90.2)	93.8 ± 7.1 (93.6-94.0)***	78.5±6.9 (78.3-78.8)	82.9±6.9 (82.6-83.2)***
**% RWC**	43.4(31.3-57.7)	47.1(33.6-63.4)***	25.1(15.9-36.2)	29.4(19.3-41.4)***	11.9(4.0-20.4)	15.4(6.6-25.4)***
**SBP (mmHg)**	115.3±10.7 (114.4-116.2)	127.8±17.5 (127.2-128.4)***	114.2±10.8 (113.7-114.8)	126.6±17.8 (126.0-127.1)***	111.0±11.3 (110.6-111.4)	123.6±19.3 (122.9-124.4)***
**DBP (mmHg)**	75.4±7.3 (74.8-76.0)	81.8±10.6 (81.4-82.2)***	72.4±7.6 (72.1-72.8)	78.8±10.7 (78.5-79.1)***	69.9±7.9 (69.6-70.2)	76.3±11.6 (75.9-76.8)***
**FPG (mg/dL)**	104(98-109)	112(104-127)***	102(97-108)	108(101-118)***	100(95-105)	105(98-113)***
**Total Cholesterol** (mg/dL)	216.1±37.2 (213.0-219.2)	217.1±43.7 (215.4-218.7)^{ns}	213.9±36.4 (212.2-215.6)	217.7±45.1 (216.4-219.1)**	208.8±35.2 (207.5-210.1)	216.1±45.8 (214.4-217.9)***
**HDLC- (mg/dL)**	60.4±12.3 (59.4-61.4)	51.3±11.8 (50.8-51.7)***	60.5±12.9 (59.9-61.1)	52.5±13.4 (52.1-52.9)***	64.6±14.2 (64.1-65.1)	56.2±15.2 (55.6-56.8)***
**LDL (mg/dL)**	136.2±33.4 (133.4-138.9)	131.3±36.2 (129.9-132.6)**	134.6±32.6 (133.1-136.2)	132.5±37.5 (131.4-133.7)*	127.1±30.9 (125.9-128.2)	131.3±36.2 (129.9-132.8)***
**TG (mg/dL)**	98(77-119)	157(113-211)***	92(71-116)	151(104-204)***	82(64-105)	121(84-179)***
**Fasting insulin (µUI/L)**	7.9(5.4-12.0)	11.8(7.7-17.8)***	5.5(3.4-8.5)	7.7(4.9-11.9)***	3.6(1.0-5.8)	5.0(2.9-7.8)***
**HOMA-IR**	2.05(1.3-3.1)	3.4(2.2-5.4)***	1.38(0.84-2.13)	2.15(1.32-3.41)***	0.89(0.27-1.43)	1.29(0.77-2.12)***
**CRP (mg/L)**	2.7(1.3-5.3)	3.0(1.5-5.7)*	1.3(0.7-2.6)	1.6(0.9-3.3)***	0.8(0.4-1.5)	1.1(0.6-2.3)***

MH- metabolically healthy**; MUH-** metabolically unhealthy; **Educational Level-** educational level; **University**- undergraduate or more; **Non-Rout, non-manual**- Non-Routine, non-manual; **Cons. alcohol-** Consumption of alcohol; High- alcohol use > 140g/week/women and >210g/week/men**; Physical act –** Physical activity**; Cons.fruit-** regular consumption of fruit ((≥5 times/week); **Cons.veget-** regular consumption of vegetables((≥5 times/week); **Reg/Poor/VeryPoor-** Regular/Poor/Very Poor; **Mental dis-** common mental disorders; **BMI**- body mass index;**%RWC-** relative weight change**; SBP-** systolic blood pressure**; DBP-** diastolic blood pressure; **FPG**- fasting plasma

glucose; **HDL-C**- high density cholesterol; **LDL**- low density cholesterol;**TG**- triglycerides; **HOMA-IR** - HOMA index ; **CRP**- C reactive protein; *p valor MH versus MUH <.05; ** p valor MH versus MUH <.01; *** p valor MH versus MUH <.001; $^{\delta}$ p valor MH versus MUH <.2; ^{ns} p valor MH versus MUH not significant

Table S6– Odds ratio [95% confidence intervals] for factors associated with being metabolically healthy according to the four criteria, among obese individuals.

**Variables/criteria**	**NHANES**<br>**OR [CI95%] (R^2 0,11)**	**NCEP**<br>**OR [CI95%] (R^2 0,09)**	**IDF**<br>**OR [CI95%] (R^2 0,09)**	**Comorbidities**<br>**OR [CI95%] (R^2 0,07)**
**Male Sex**	2.63[2.10-3.29]***	2.23[1.85-2.69]***	2.86[2.35-3.48]***	2.53[2.02-3.16]***
**Age**	0.95[0.93-0.96]***	0.95[0.94-0.96]***	0.95[0.94-0.96]***	0.95[0.94-0.96]***
**Educational level**				--
Elementary school	0.53[0.34-0.83]**	0.61[0.42-0.88]**	0.56[0.38-0.82]**	
High school	0.88[0.68-1.15]	0.92[0.73-1.16]	0.89[0.70-1.12]	
University degree	1.0	1.0	1.0	
**Occupation**				
Non-rout., non-manual	1.0	1.0	1.0	1.0
Routine non-manual	0.92[0.66-1.27]	0.81[0.61-1.06]	0.87[0.65-1.16]	1.10[0.81-1.48]
Manual	0.87[0.60-1.25]	0.91[0.67-1.24]	0.98[0.71-1.35]	1.36[1.03-1.79]*
**Smoking**				--
Never	1.0	1.0	1.0	
Former	0.73[0.58-0.92]**	0.73[0.60-0.88]**	0.77[0.63-0.94]*	
Current	0.65[0.46-0.91]**	0.69[0.52-0.92]*	0.68[0.51-0.91]*	
**Physical activity**				
High	1.0	1.0	1.0	1.0
Moderate	0.83[0.52-1.33]	0.62[0.42-0.94]*	0.65[0.43-0.98]*	0.55[0.34-0.87]*
Low	0.73[0.50-1.09]	0.58[0.42-0.82]**	0.60[0.42-0.85]**	0.57[0.39-0.84]**
**Poor self-rated health**	0.63[0.50-0.80]***	0.61[0.51-0.75]***	0.63[0.51-0.77]***	0.60[0.48-0.76]***
**Fruit consumption**	--	--	--	0.78[0.64-0.95]*
**BMI**	0.89[0.86-0.93]***	0.91[0.88-0.94]***	0.94[0.91-0.97]***	0.94[0.91-0.98]**
**%RWC**	0.99[0.98-0.99]**	0.99[0.991-0.999]*	0.99[0.990-0.998]*	0.99[0.99-1.00]

OR- Odds ratio, [95%CI]- 95% confidence intervals, R^2- Pseudo R^2, Non-rout., non-manual- Non-routine, non-manual. Poor Self-rated health - regular/poor and very poor self-rated health, BMI- body mass index, %RWC relative weight change, *p< .05, **p<.01, *** p<.001. NHANES- National Health Examination Surveys; NCEP- National Cholesterol Education Panel; IDF- International Diabetes Federation. Final logistic

model- References- Sex- female; Age- continuous; Education level- university degree; Occupation- Non-routine, non-manual; Smoking- never; Physical activity- highly active; Self rated health- good/very good; Fruit consumption (≥5 times/week as reference), BMI- body mass index- continuous; % RWC- relative weight change- continuous.

Table S7– Odds ratio [95% confidence intervals] for factors associated with being metabolically healthy according to the four criteria, among overweight individuals

**Variables/criteria**	**NHANES**<br>**OR [CI95%] (R^2 0.11)**	**NCEP**<br>**OR [CI 95%] ($R^2$0.11)**	**IDF**<br>**OR [CI 95%] (R^2 0.11)**	**Comorbidities**<br>**OR [CI 95%] (R^2 0.09)**
**Male Sex**	2.31[2.01-2.65]***	--	1.61[1.42-1.83]***	1.94[1,70-2.22]***
**Age**	0.93[0.93-0.94]***	0.95[0.94-0.96]***	0.95[0.94-0.95]***	0.94[0.94-0.95]***
**Civil status- no married**	1.15[1.01-1.32]*	--	--	--
**Occupation**				
Non-rout., non-manual	1.0	1.0	1.0	1.0
Routine non-manual	0.93[0.71-1.21]	1.14[0.89-1.47]	0.95[0.74-1.23]	0.96[0.73-1.27]
Manual	0.90[0.67-1.22]	1.20[0.91-1.58]	0.92[0.70-1.22]	0.97[0.72-1.31]
**Premature birth**	--	--	0.70[0.53-0.92]*	--
**Smoking**			--	
Never	1.0	1.0		1.0
Former	0.88[0.77-1.00]	0.78[0.68-0.89]***		0.92[0.80-1.06]
Current	0.78[0.64-0.95]*	0.72[0.59-0.87]**		0.64[0.52-0.78]***
**Alcohol consumption**				--
No	1.0	1.0	1.0	
Moderate	1.03[0.89-1.18]	1.01[0.88-1.17]	1.01[0.88-1.16]	
High	0.79[0.60-1.02]	0.68[0.53-0.85]**	0.63[0.50-0.80]***	
**Physical activity**				
High	1.0	1.0	1.0	1.0
Moderate	0.70[0.54-0.90]**	0.63[0.48-0.82]**	0.60[0.47-0.77]***	0.70[0.54-0.90]**
Low	0.68[0.55-0.84]***	0.57[0.45-0.72]***	0.57[0.46-0.70]***	0.74[0.60-0.91]**
**Social class**				
Low	0.74[0.56-0.98]*	1.04[0.79-1.37]	0.78[0.59-1.02]	0.70[0.52-0.94]*
Middle	0.81[0.68-0.97]*	0.86[0.72-1.04]	0.79[0.67-0.94]**	0.79[0.67-0.95]*
High	1.0	1.0	1.0	1.0
**Poor Self-rated health**	0.61[0.51-0.72]***	0.59[0.51-0.68]***	0.56[0.48-0.66]***	0.55[0.46-0.66]***

**BMI**	0.84[0.81-0.88]***	0.72[0.69-0.75]***	0.74[0.71-0.78]***	0.86[0.82-0.90]***
**%RWC**	0.99[0.98-0.99]***	0.98[0.98-0.99]***	0.98[0.98-0.99]***	0.98[0.98-0.99]***

OR- Odds ratio, [95%CI]- 95% confidence intervals, R^2- Pseudo R^2, Non-rout., non-manual- Non-routine., non-manual; Poor Self-rated health - regular/poor and very poor self-rated health, BMI- body mass index, %RWC relative weight change, *p<.05, **p<.01, *** p<.001. NHANES- National Health Examination Surveys ; NCEP- National Cholesterol Education Panel; IDF- International Diabetes Federation. Final logistic model- References- Sex- female; Age- continuous; Civil status- married; Occupation- Non-routine,non-manual;Smoking- never; Alcohol consumption- no use; Physical activity- highly active; Social class- high; Self rated health- good/very good; BMI- body mass index-continuous; % RWC- relative weight change- continuous.

Table S8 – Odds ratio [95% confidence intervals] for factors associated with being metabolically healthy according to the four criteria, among normal weight individuals

**Variables/criteria**	**NHANES**<br>**OR [CI95%] (R^2 0.13)**	**NCEP**<br>**OR [CI 95%] ($R^2$0.11)**	**IDF**<br>**OR [CI 95%] (R^2 0.13)**	**Comorbidities**<br>**OR [CI 95%] (R^2 0.11)**
**Sex**	2.14[1.87-2.45]***	1.86[1.52-2.27]***	--	1.68 [1.47-1.92]***
**Age**	0.93[0.92-0.94]***	0.94[0.93-0.95]***	0.94[0.93-0.95]***	0.94[0.93-0.95]***
**Occupation**				
Non-rout.,non-manual	1.0	1.0	1.0	1.0
Routine non-manual	1.00[0.76-1.34]	0.73[0.50-1.08]	0.73[0.52-1.01]	0.91[0.68-1.21]
Manual	1.11[0.81-1.51]	0.89[0.59-1.36]	0.87[0.60-1.25]	0.96[0.70-1.32]
**Smoking**	--	--	--	
Never				1.0
Former				1.07[0.92-1.25]
Current				0.79[0.65-0.95]*
**Alcohol consumption**		--		
No	1.0		1.0	1.0
Moderate	1.13[0.97-1.29]		1.12 [0.93-1.35]	1.19[1.04-1.38]*
High	0.69[0.52-0.92]*		0.67[0.48-0.94]*	0.68[0.51-0.91]*
**Physical activity**	--	--		
High			1.0	1.0
Moderate			0.80[0.56-1.14]	0.97[0.76-1.24]
Low			0.69[0.51-0.94]*	0.84[0.69-1.03]
**Social class**				
Low	0.69[0.52-0.92]*	0.49[0.33-0.74]**	0.56[0.39-0.79]*	0.64[0.48-0.86]**
Middle	0.74[0.62-0.88]**	0.64[0.48-0.85]**	0.73[0.57-0.92]**	0.72[0.60-0.85]***
High	1.0	1.0	1.0	1.0
**Poor Self-rated health**	0.69[0.58-0.84]***	--	0.76[0.61-0.96]*	0.59[0.49-0.71]***
**Mental disorders (yes)**	--	0.72[0.58-0.90]**	0.81[0.67-0.98]*	--
**BMI**	0.83[0.80-0.87]***	0.74[0.69-0.79]***	0.66[0.62-0.71]***	0.85[0.82-0.89]***

**%RWC**	0.98[0.98-0.99]***	0.98[0.98-0.99]***	0.98[0.98-0.99]***	0.98[0.98-0.99]***

OR- Odds ratio, [95%CI]- 95% confidence intervals, R^2- Pseudo R^2, Non-rout.,non-manual- Non-routine.,non-manual; Poor Self-rated health – regular, poor or very poor self-rated health ,BMI- body mass index, %RWC relative weight change, *p< .05, **p< .01, ***p< .001. NHANES- National Health Examination Surveys ; NCEP- National Cholesterol Education Panel; IDF- International Diabetes Federation. Final logistic model- References- Sex- female; Age- continuous; Occupation- Non-routine, non-manual Alcohol consumption- no use; Physical activity- highly active; Social class- high; Self rated health- good/very good; Mental disorders- common mental disorders- no; BMI- body mass index-continuous; %RWC- relative weight change(continuous).
